# Glycerol Monocaprylate Modulates Gut Microbiota and Increases Short-Chain Fatty Acids Production without Adverse Effects on Metabolism and Inflammation

**DOI:** 10.3390/nu13051427

**Published:** 2021-04-23

**Authors:** Junhui Zhang, Fengqin Feng, Minjie Zhao

**Affiliations:** 1 College of Biosystems Engineering and Food Science, Zhejiang University, Hangzhou 310058, China; zhangjunhui9916@163.com (J.Z.); feng_fengqin@hotmail.com (F.F.); 2 National Engineering Laboratory of Intelligent Food Technology and Equipment, Zhejiang University, Hangzhou 310058, China; 3 Key Laboratory for Agro-Products Postharvest Handling of Ministry of Agriculture and Rural Affairs, Zhejiang University, Hangzhou 310058, China; 4 Key Laboratory for Agro-Products Nutritional Evaluation of Ministry of Agriculture, Zhejiang University, Hangzhou 310058, China; 5 Ningbo Research Institute, Zhejiang University, Ningbo 315100, China

**Keywords:** glycerol monocaprylate, gut microbiota, short-chain fatty acids, glucose and lipid metabolism, inflammation

## Abstract

Glycerol monocaprylate (GMC) is a glycerol derivative of medium-chain fatty acids (MCFAs) and is widely used as a preservative in food processing. However, GMC and its hydrolytic acid (octylic acid) have antibacterial properties that may affect the physiology and intestinal microecology of the human body. Therefore, in this study, the effects of two different dosages of GMC (150 and 1600 mg kg^−1^) on glucose, lipid metabolism, inflammation, and intestinal microecology of normal diet-fed C57BL/6 mice were comprehensively investigated. The obtained results showed that the level of triglycerides (TGs) in the low-dose group down-regulated significantly, and the anti-inflammatory cytokine interleukin 10 (IL-10) significantly increased, while the pro-inflammatory cytokines monocyte chemotactic protein 1 (MCP-1) and interleukin 1beta (IL-1β) in the high-dose group were significantly decreased. Importantly, GMC promoted the α-diversity of gut microbiota in normal-diet-fed mice, regardless of dosages. Additionally, it was found that the low-dose treatment of GMC significantly increased the abundance of Lactobacillus, while the high-dose treatment of GMC significantly increased the abundance of SCFA-producers such as Clostridiales, Lachnospiraceae, and *Ruminococcus*. Moreover, the content of short-chain fatty acids (SCFAs) was significantly increased by GMC supplementation. Thus, our research provides a novel insight into the effects of GMC on gut microbiota and physiological characteristics.

## 1. Introduction

Glycerol monocaprylate (GMC) is a common medium-chain fatty acid monoglyceride synthesized by 1:1 caprylic acid and glycerol. Due to its antibacterial properties, GMC is regarded as a new type of non-toxic and highly effective broad-spectrum food preservative by the National Health Commission of the People’s Republic of China. Moreover, GMC has excellent emulsifying properties, which are helpful for stabilizing food shape, improving tissue structure, and optimizing product quality [1]. GMC with these effective antibacterial and emulsifying properties has been widely used in a variety of foods, such as wet-fresh noodles, pastry, and meat [2].

MCFAs (e.g., caprylic acid, capric acid, and lauric acid) have distinct metabolic merits, as they can be absorbed into the portal vein through the gut and then transported directly to the liver to be rapidly metabolized and no longer synthesize triglycerides. Importantly, in hepatocytes, MCFAs are carried to the mitochondria for β-oxidation without carnitine transferase dependence [3,4]. Consequently, they are not stored and thus have less burden on liver metabolism. Due to their special metabolism characteristics, MCFAs can increase energy consumption and fat oxidation, decrease blood triglycerides, improve lipoprotein metabolism, reduce the weight of patients with hypertriglyceridemia, and prevent obesity [5]. For instance, Li et al. found that caprylic acid fed to mice could lower body weight and decrease the levels of total cholesterol and low-density lipoprotein cholesterol [6]. GMC, as a typical glycerol derivative of MCFAs, could be hydrolyzed to caprylic acid and glycerol by lipase in the intestine. It is known that GMC has a long residence time in the intestinal tract, and both GMC and its hydrolytic acid (octylic acid) have antibacterial properties [7]. Hence, GMC may significantly influence the gut microbiota. Nevertheless, its exact effects on host metabolism are still unclear and need further study.

Recently, accumulating evidence has suggested that gut microbiota and its metabolites could have an important effect as a bridge between the diet and the host and subsequently regulate human health [8,9]. For instance, microbes in the intestines can ferment indigestible dietary fibers and resistant starch to produce short-chain fatty acids (SCFAs). SCFAs can regulate immune responses and energy metabolism in vivo by inhibiting histone deacetylases and also activating G protein-coupled receptors [10]. Fushimi et al. reported that intake of dietary acetic acid could promote bile acid excretion, inhibit lipid synthesis in the liver, and reduce the level of serum cholesterol and triglycerides (TG) in cholesterol-fed rats [11]. Kelly et al. demonstrated that the intestinal epithelium absorbs butyrate to correct physiological hypoxia and maintain intestinal barrier function by restoring hypoxia inducible factor expression [12]. Furthermore, intestinal microorganisms play a fundamental role in various physiological activities of the host, such as digestion and absorption of substances, carbohydrate metabolism, fat metabolism, amino acid metabolism, immune regulation, etc. [13]. Thus, maintaining a healthy and stable intestinal microbiome is of great importance. 

In our earlier research on glycerol monolaurate (GML), it was found that adding a low-dose of GML (150 mg kg^−1^) in a normal diet promoted metabolic syndrome, mild inflammation, and dysbiosis of gut microbiota [14]. A further study by Zhao et al. showed that using a high dose of GML (1600 mg kg^−1^) alleviated high-fat diet-induced metabolic disorders and the dysbiosis of gut microbiota, while reducing levels of serum proinflammatory cytokines [15]. However, it is unclear whether GMC has certain effects on metabolism, inflammation, and gut microbiota. Therefore, in the current study, the effects of low-dose (150 mg kg^−1^) and high-dose (1600 mg kg^−1^) GMC on physiology and intestinal microecology in normal-diet-fed mice were extensively investigated. The results demonstrated that the addition of GMC effectively improved the composition and structure of gut microbiota, promoted the abundance of Lactobacillus in the 150 mg kg^−1^ treated group, and increased the abundance of SCFA-producers such as Clostridiales, Lachnospiraceae, and *Ruminococcus* in the 1600 mg kg^−1^ treated group without adverse effects on metabolism and inflammation. Our findings provide a novel insight into the potential mechanisms of GMC’s physiological action by demonstrating modulation of gut microbiota populations and activities, including the promotion of microbial diversity and the growth of beneficial bacteria as well as the stimulation of SCFA production.

## 2. Materials and Methods

### 2.1. Animals and Diet

Wild-type male C57BL/6 mice (4–5 weeks of age) were purchased from Shanghai SLAC Laboratory Animal Co., Ltd. (Shanghai, China) and then bred and housed at Zhejiang Chinese Medical University Laboratory Animal Research Center (Hangzhou, China) under institutionally approved protocols (Institutional Animal Ethics Committee no. 11164). Mice were maintained at 24 °C with a 12 h light/dark cycle, with free access to standard rodent chow diet and water. After a week of acclimatization on a regular diet, mice were divided randomly into three groups (*n* = 12 per group, four mice per cage): (1) NCD, normal chow diet (Shanghai Fan Bo Co., Ltd., Shanghai, China) as the control group; (2) GMC150, the normal chow diet incorporating 150 mg kg^−1^ GMC as the low-dosage treatment group; (3) GMC1600, the normal chow diet incorporating 1600 mg kg^−1^ GMC as the high-dosage treatment group. The diet of the GMC group was made by crushing the control diet and adding the GMC and then pelletizing. The GMC was purchased from Henan Zhengtong Food Technology Co., Ltd. The feeding process took 22 weeks, in which body weight and dietary intake were measured each week. Furthermore, each mouse was placed in a separate metabolic cage and its feces were collected within 2 min after defecation. Then the fresh fecal was immediately stored at –80 °C for downstream analysis. Fecal collection was conducted once a month. After the GMC treatment was completed, the mice fasted for 12 h, and blood was drawn from the intraorbital retrobulbar capillary plexus. Then, mice were euthanized by carbon dioxide inhalation, and the livers, epididymal tissue, and brown fat tissue (BAT) were collected, weighed, and stored at −80 °C for downstream analysis.

### 2.2. Glucose Metabolism

At week 20, an intravenous glucose tolerance test was performed as follows: eight randomly selected mice from each group fasted for 12 h and then were injected intraperitoneally with 2 g kg^−1^ body weight glucose solution. Finally, blood samples were collected from the tail vein at the designated time points (0, 30, 60, 90, and 120 min), and the glucose concentrations were measured using an Accu-Check glucometer (Roche Diagnostics Deutschland GmbH, Mannheim, Germany).

Fasting serum glucose concentration was measured with a commercially available kit (Nanjing Jiancheng, Nanjing, China), and fasting serum insulin was measured by a mouse ELISA kit (Wuhan ColorfulGene Biological Technology Co., Ltd., Wuhan, China). Next, the model assessment of insulin resistance homeostasis (HOMA-IR) was computed with the following formula: serum insulin (µMm L^−1^) × serum glucose (mmol L^−1^)/22.5.

### 2.3. Biochemical Analysis

The concentration of serum total cholesterol (T-CHO), triglycerides (TGs), low-density lipoprotein cholesterol (LDL-C), and high-density lipoprotein cholesterol (HDL-C) were quantified by commercially available kits (*n* = 12 per group) from Nanjing Jiancheng Bioengineering Institute, following the respective manufacturer’s instructions.

Serum free fatty acid (FFA), leptin (LEP), peptide YY (PYY), adiponectin (ADP), glucagon-like peptide 1 (GLP-1), lipopolysaccharide binding protein (LBP), lipopolysaccharide (LPS), interleukin 10 (IL-10), interleukin 6 (IL-6), interleukin 1β (IL-1β), tumor necrosis factor (TNF), and monocyte chemotactic protein 1 (MCP-1) were measured with an ELISA kit (Wuhan ColorfulGene Biological Technology Co., Ltd., Wuhan, China) under the respective manufacturer’s instructions (*n* = 8 per group; serum samples from mice were randomly selected).

### 2.4. H&E Staining and Histology Analysis

After euthanasia, using 10% buffered formalin fixed mouse liver and epididymal adipose tissue at room temperature for 24 h, the fixed tissue of each mouse (*n* = 12 per group) was embedded in paraffin, sliced at 5 µm thickness, and stained with hematoxylin and eosin (H&E) according to standard protocols. The size and number of stained epididymal fat tissue were analyzed by Image-Pro Plus 6.1 (Media Cybernetics, Inc., Rockville, MD, USA).

### 2.5. Gene Expression Analysis by Quantitative Real-Time PCR (qRT-PCR)

Using TRIzol reagent (Invitrogen, Carlsbad, CA, USA) total RNAs of the liver and brown fat were isolated (*n* = 6 group; samples from mice were randomly selected). cDNA was obtained by reverse transcription of total RNAs with HiScript Reverse Transcription kit (Vazyme, Jiangsu, China). The qPCR was conducted with the 2×ChamQ SYBR Color qPCR Master Mix (Vazyme Biotech Co., Ltd., Nanjing, China) on a LightCycler 480 system (Roche Applied Science, Indianapolis, IA, USA) with specific mouse primers. Results were normalized to the housekeeping YWHAZ gene and calculated based on the 2^−∆∆Ct^ method. All the primer sequences are shown in Supplemental Table S1.

### 2.6. Western Blot

Total protein of the liver was extracted with the Tissue Protein Extraction Reagent (Thermo Pierce, Rockford, IL, USA) and quantified with the BCA quantitative kit (Beyotime, Nantong, China). The protein was mixed thoroughly with the loading buffer at the ratio of 1:3 and heated at 100 °C for 10 min to denature the protein. The protein was centrifuged at 3000 rpm for 4 min and separated using 12% SDS-PAGE gel. It was then transferred onto a PVDF membrane and blocked with 8% nonfat milk. The membrane was first incubated with primary antibodies against β-actin (1:10,000 dilution), PPARγ2 (1:1000 dilution), GCK (1:1000 dilution), PPARα (1:1000 dilution), and FOXO1 (1:1000 dilution) at 4 °C for 12 h and then with the secondary antibody for 1 h. The optical density of the strips was analyzed with Image J software, and the values were normalized to β-actin. 

### 2.7. Gut Microbiota Analysis with 16S rRNA Gene Sequencing

Eight mice from each group were randomly selected for 16S rRNA gene sequencing. The total microbial DNA was isolated from weighed feces, which were collected at week 21 using the QIAamp DNA Stool Mini Kit (Qiagen, Dusseldorf, Germany) following the manufacturer’s protocols. The bacterial 16S rRNA genes, hypervariable regions V3–V4, were amplified with the forward primer and the reverse primer by the thermocycler PCR system (Gene Amp 9700, ABI, Waltham, MA, USA). The amplicons recovered from 2% agarose gel were purified with AxyPrep DNA Gel Extraction Kit (Axygen Biosciences, Union City, CA, America), quantified by QuantiFluor™-ST (Promega, Madison, WI, USA), and then paired-end sequenced (2 × 300) on an Illumina MiSeq platform (Illumina, San Diego, CA, USA) following the standard procedure by Majorbio Bio-Pharm Technology Co. Ltd. (Shanghai, China). Then, raw reads from the original DNA fragments were quality-filtered and merged. Sequences were clustered into the same operational taxonomic units (OTUs) with the UPARSE software package. Then, the sequence was analyzed by the RDP Classifier algorithm against the Greengenes 16S rRNA bacteria database. The species abundance of each sample was counted at the phylum level as well as the family level and studied visually through the histogram visualization method. A heatmap based on the top 30 dominant genera was used to present the Community species composition and species abundance information. Linear discriminant analysis effect size (LEfSe) analysis was performed to analyze the species differences between the groups. Finally, the correlation heatmap was applied to visualize the relationship between different species in the sample and blood biochemical criterion and to evaluate the correlation between microbial classification and the blood biochemical criterion.

### 2.8. Short-Chain Fatty Acids (SCFAs) Composition Analysis

The composition and concentration of SCFAs were quantified from frozen fecal samples using gas chromatography. A total of 50 mg of feces was weighed in a centrifuge tube to which 250 µL of ultrapure water was added (*n* = 8 per group; feces from mice were randomly selected). Then the suspension was vortexed for 5 min, and 10 µL of 5 mol L^−1^ HCl was added into the centrifuge tube and vortexed again for 1 min. The suspension was then incubated for 10 min at room temperature, with intermittent shaking. Prior to chromatographic analysis, the fecal suspension was centrifuged at 12,000 rpm for 20 min. Then, 200 µL of supernatant was transferred to a new centrifuge tube and supplemented with 2-ethylbutyric acid to 1 mmol L^−1^. Afterward, 1 µL of injection solution was analyzed using a capillary gas chromatograph (GC-2014, SHIMADZU, Kyoto, Japan) with the column DB-FFAP (J&W Scientific, Agilent Technologies Inc., Santa Clara, CA, USA). The specific operation parameters were as follows: flame ionization detector, 240 °C; injection port, 200 °C; temperature raising program: 100 °C for 30 s, 8 °C min^−1^ until 180 °C (1 min), 20 °C min^−1^ until 200 °C (15 min); nitrogen, hydrogen, and air flow rate: 20, 30 and 300 mL min^−1^, respectively.

### 2.9. Statistical Analysis

The results were expressed as mean ± SEM, and statistical analysis, as well as the drawing, were performed by GraphPad Prism 6.0 (GraphPad Software Inc., San Diego, CA, USA). Significance among groups was determined using one-way analysis of variance (ANOVA) with Tukey’s multiple comparison post-tests. A *p*-value < 0.05 was considered statistically significant.

## 3. Results

### 3.1. Effects of GMC Supplementation on Body Weight, Feed Intake, Adipocyte Size, and Liver Histology 

**Figure 1:**
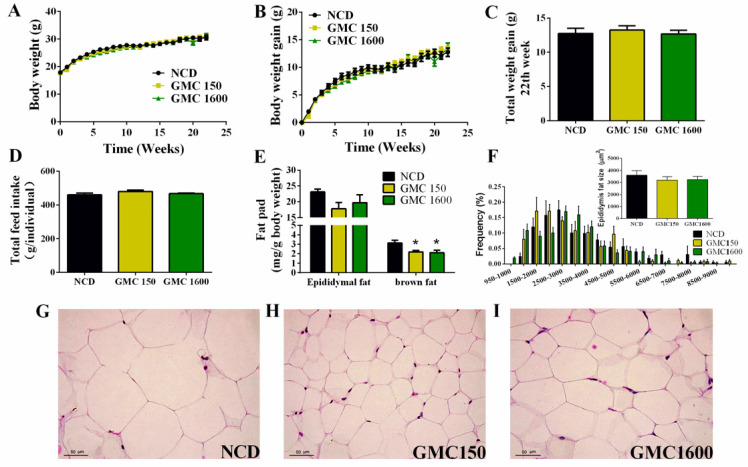
Effects of GMC treatment on (**A**) original body weight change in each group, (**B**) body weight gain, (**C**) total weight gain in the 22nd week, (**D**) total feed intake, and (**E**) the relative weight of epididymal fat pad and brown fat tissue of mice. (**F**–**I**) Epididymal adipocyte size and frequency calculated by H&E staining and estimated with the Image-Pro software (200×). Data are expressed as mean ± SEM, *n* = 12 (* *p* < 0.05 vs. NCD).

Compared with the NCD group, low-dose treatment and high-dose treatment of GMC had no significant effect on body weight (Figure 1A–C). There were no significant changes in total feed intake among the NCD and GMC groups (Figure 1D). No obvious differences were observed in the relative weight of epididymal adipose tissues among different groups. However, the relative brown adipose tissue (BAT) weight to body weight ratio at 150 mg kg^−1^ and 1600 mg kg^−1^ GMC supplementation showed a significant decrease compared to the NCD group (*p* = 0.0280 and *p* = 0.0161, respectively, Figure 1E). Consistent with the result of the relative weight of epididymal adipose, the frequency of 950–9500 μm^2^ of stained fat droplets and the size of epididymal adipocyte had no significant differences between the NCD and GMC groups (Figure 1F–I). Furthermore, no abnormalities were found in the H&E histology of the liver tissues (Supplemental Figure S1A–C).

### 3.2. Effects of GMC Supplementation on Glucose and Lipid Metabolism

**Figure 2:**
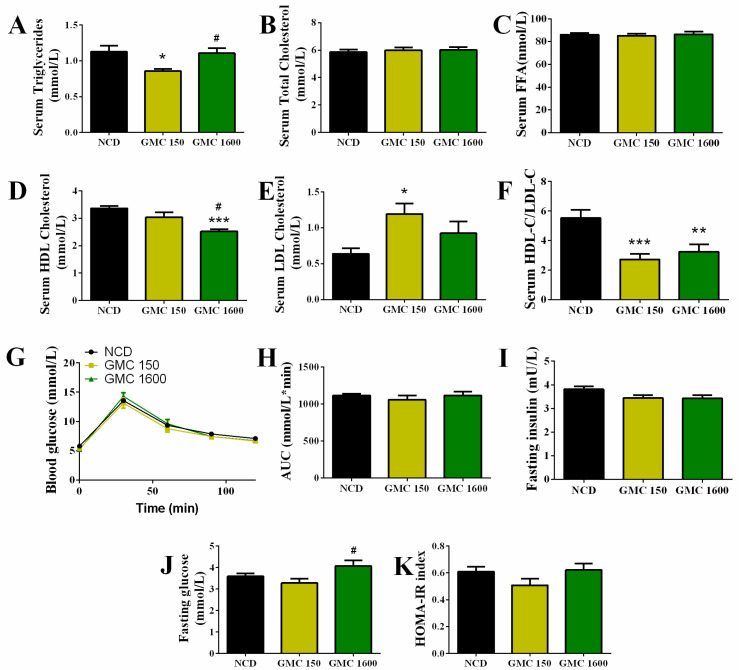
Changes in the glucose and lipid metabolism. (**A**) Triglyceride (TG), (**B**) Total cholesterol (T-CHO), (**C**) Free fatty acid (FFA), (**D**) High-density lipoprotein cholesterol (HDL-C), (**E**) Low-density lipoprotein cholesterol (LDL-C), (**F**) the ratio of HDL-C to LDL-C, (**G**) glucose tolerance test (IGTT), (**H**) area under the curve (AUC) of IGTT, (**I**) fasting insulin, (**J**) fasting glucose, and (**K**) HOMA-IR. Data are expressed as mean ± SEM, *n* = 8–12 (* *p* < 0.05 vs. NCD, ** *p* < 0.01 vs. NCD, *** *p* < 0.001 vs. NCD, # *p* < 0.05 vs. GMC150).

Low-dose treatment of GMC statistically reduced the level of serum TG when compared with the NCD group and 1600 mg kg^−1^ GMC supplementation group (*p* = 0.0146 and *p* = 0.0255, respectively, Figure 2A). Referring to the content of T-CHO and FFA in the serum, no obvious differences were found among various groups (Figure 2B,C). Markedly, adding 1600 mg kg^−1^ GMC into the diet significantly decreased the content of HDL-C in comparison to the NCD group (*p* < 0.001, Figure 2D). In contrast, the concentration of LDL-C in the 150 mg kg^−1^ GMC group was significantly up-regulated when compared with the NCD group (*p* = 0.0167, Figure 2E). Furthermore, both the 150 mg kg^−1^ GMC and 1600 mg kg^−1^ GMC supplementation resulted in a marked decline in the ratio of HDL-C to LDL-C in contrast with the NCD group (*p* < 0.001, *p* = 0.0053, respectively, Figure 2F). To evaluate the influence of different dosages of GMC supplementation on glucose metabolism, an intraperitoneal glucose tolerance test (IGTT) was conducted. There was no detectable change observed in the blood glucose curve and the area under the curve (AUC) among different groups (Figure 2G,H). In addition, 1600 mg kg^−1^ GMC supplementation resulted in a decreasing tendency in the concentration of serum fasting insulin compared to the NCD group (*p* = 0.0977, Figure 2I). Meanwhile, the 1600 mg kg^−1^ GMC treatment significantly increased the level of serum fasting glucose in comparison with 150 mg kg^−1^ GMC treatment (*p* = 0.0362, Figure 2J). The HOMA-IR of the GMC and NCD groups showed no obvious differences (Figure 2K).

### 3.3. Effects of GMC Supplementation on Appetite-Hormone Level and Inflammation-Related Cytokines in Serum

**Figure 3:**
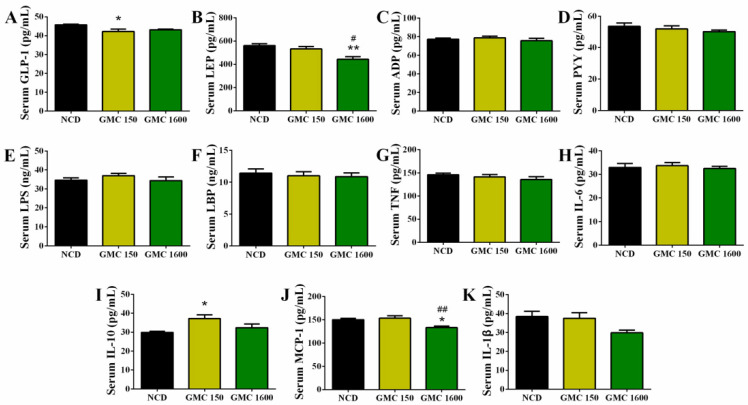
Changes in serum hormone levels and inflammation-related cytokines. (**A**) Glucagon-like peptide 1 (GLP-1), (**B**) Leptin (LEP), (**C**) Adiponectin (ADP), (**D**) Peptide YY (PYY), (**E**) Lipopolysaccharide (LPS), (**F**) Lipopolysaccharide binding protein (LBP), (**G**) Tumor necrosis factor (TNF), (**H**) Interleukin 6 (IL-6), (**I**) Interleukin 10 (IL-10), (**J**) Monocyte chemotactic protein 1 (MCP-1), and (**K**) Interleukin 1β (IL-1β). Data are expressed as mean ± SEM, *n* = 8 (* *p* < 0.05 vs. NCD, ** *p* < 0.01 vs. NCD, # *p* < 0.05 vs. GMC150, ## *p* < 0.01 vs. GMC150).

Adding 150 mg kg^−1^ GMC into the diet significantly reduced the concentration of the serum GLP-1 (*p* = 0.0128, Figure 3A). Interestingly, the 1600 mg kg^−1^ GMC treatment had an obvious down-regulation in the concentration of LEP compared to the NCD group and low-dose treatment group (*p* = 0.0013 and *p* = 0.0135, respectively). However, there were no significant differences in serum ADP, PYY, LPS, LBP, TNF, and IL-6 among different groups (Figure 3C–H). Importantly, the content of serum IL-10 had a significant up-regulation after the low-dose treatment of GMC (*p* = 0.0111, Figure 3I). In contrast with the NCD group and 150 mg kg^−1^ GMC treated group, the concentration of serum MCP-1 in the 1600 mg kg^−1^ GMC group had an obvious down-regulation (*p* = 0.0182 and *p* = 0.0050, respectively, Figure 3J). Moreover, the level of serum IL-1β in the 1600 mg kg^−1^ GMC group decreased compared with the NCD group (*p* = 0.0601, Figure 3K).

### 3.4. Effects of GMC Supplementation on the Expressions of Genes and Proteins Related with Glucose and Lipid Metabolism and Inflammation

**Figure 4:**
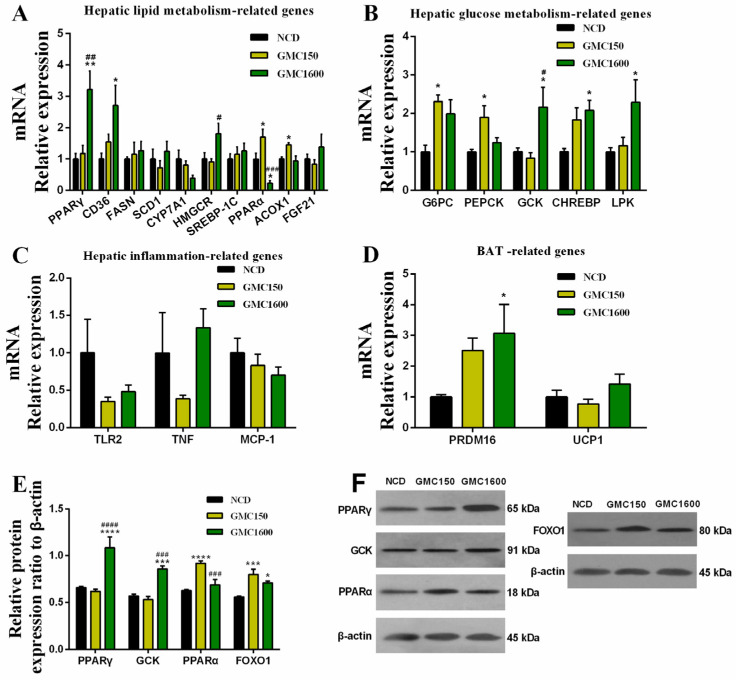
Changes in the expressions of genes and protein related with glucose and lipid metabolism and inflammation. (**A**) Hepatic lipid metabolism-related genes: PPARγ2, CD36, FASN, SCD1, CYP7A1, HMGCR, SREBP-1C, PPARα, ACOX1, and FGF21; (**B**) hepatic glucose metabolism-related genes: G6PC, PEPCK, GCK, CHREBP, and LPK; (**C**) hepatic inflammation-related genes: TLR2, TNF, and MCP-1; (**D**) BAT-related genes: PRDM16 and UCP1; (**E**,**F**) relative expression of the liver: PPARγ2, GCK, PPARα, and FOXO1. Data are expressed as mean ± SEM, *n* = 6 (* *p* < 0.05 vs. NCD, ** *p* < 0.01 vs. NCD, *** *p* < 0.001 vs. NCD, **** *p* < 0.0001 vs. NCD, # *p* < 0.05 vs. GMC150, ## *p* < 0.01 vs. GMC150, ### *p* < 0.001 vs. GMC150, #### *p* < 0.0001 vs. GMC150).

The mRNA and protein expression of PPARα was significantly increased by 150 mg kg^−1^ GMC treatment compared with the NCD group (*p* = 0.0483, Figure 4A and *p* < 0.0001, Figure 4E). Interestingly, the expression of ACOX1, which is the target gene of PPARα, also up-regulated in the 150 mg kg^−1^ GMC treated group (*p* = 0.0323, Figure 4A). Moreover, an obvious up-regulation of the mRNA and protein expression of PPARγ2 was observed when adding 1600 mg kg^−1^ GMC into the diet (*p* = 0.0019, Figure 4A and *p* < 0.0001, Figure 4E). Meanwhile, CD36, which is the target gene of PPARγ2, also had a higher expression in the 1600 mg kg^−1^ GMC group (*p* = 0.0253, Figure 4A). However, there were no significant changes in the expression of FASN, SCD1, CYP7A1, SREBP-1C, and FGF21 in different groups (Figure 4A). The mRNA level of HMGCR under the 1600 mg kg^−1^ GMC treatment was higher than that in the low-dose treatment group (*p* = 0.0367, Figure 4A). Interestingly, G6PC and PEPCK, which are associated with the glucose metabolism, significantly up-regulated in the 150 mg kg^−1^ GMC treatment group (*p* = 0.0105 and *p* = 0.0427, respectively, Figure 4B). The mRNA and protein expression of GCK were significantly increased in the 1600 mg kg^−1^ GMC group (*p* = 0.0487, Figure 4B and *p* < 0.0001, Figure 4E). Moreover, the expression of CHREBP and its target gene LPK were significantly increased by 1600 mg kg^−1^ GMC treatment (*p* = 0.0178 and *p* = 0.0267, respectively, Figure 4B). Interestingly, the protein expression of FOXO1 significantly increased after GMC treatment (*p* < 0.001 and *p* = 0.0469, respectively, Figure 4E). Furthermore, inflammation-related genes, including TLR2, TNF, and MCP-1, had a down-regulation in the GMC-treated groups (Figure 4C). Notably, the expression of PRDM16, which is associated with the thermogenesis of brown fat tissue (BAT), was significantly increased in the 1600 mg kg^−1^ GMC group (*p* = 0.0451, Figure 4D). The expression of UCP1 had no significant changes in the different groups (Figure 4D).

### 3.5. Effects of GMC Supplementation on the Diversity and Composition of Gut Microbiota

**Figure 5:**
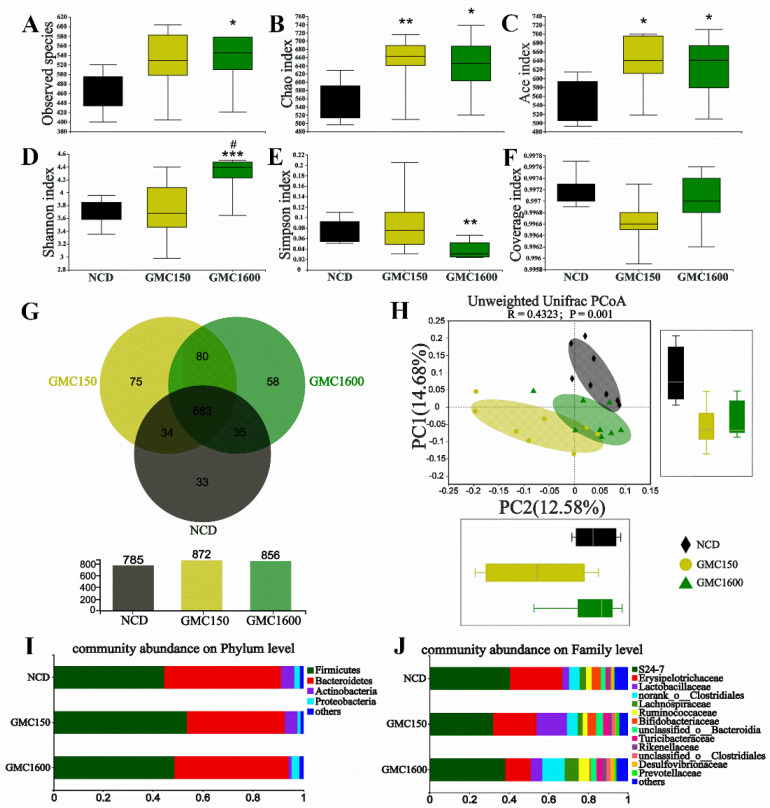
GMC supplementation altered the structure of intestinal microbiota. α-diversity: (**A**) observed species, (**B**) Chao index, (**C**) Ace index, (**D**) Shannon index, (**E**) Simpson index, (**F**) Coverage index, and (**G**) OTU Venn diagram between treatments; ꞵ-diversity: (**H**) PCoA plot based on unweighted UniFrac distances. Relative abundance of gut microbiota at the phylum level (**I**) and at family level (**J**). Data are expressed as mean ± SEM, *n* = 8 (* *p* < 0.05 vs. NCD, ** *p* < 0.01 vs. NCD, *** *p* < 0.001 vs. NCD, # *p* < 0.05 vs. GMC150).

For α-diversity analysis, the number of observed species in the 150 mg kg^−1^ GMC group was higher than in the NCD group (Figure 5A). Additionally, the Chao index and the Ace index were significantly increased by 150 mg kg^−1^ GMC treatment (*p* = 0.0074 and *p* = 0.0181, respectively, Figure 5B,C). Importantly, 1600 mg kg^−1^ GMC presented an obvious up-regulation in observed species, the Chao index, and the Ace index (*p* = 0.0178, *p* = 0.0313, and *p* = 0.0483, respectively) as well as an obvious down-regulation in Simpson index compared with the NCD group (*p* =0.0101, Figure 5A–E). The results of the coverage index indicated that the real situation of microbes in the samples was reflected (Figure 5F). Interestingly, 785, 872, and 856 bacterial OTUs were obtained in the NCD, 150 mg kg^−1^, and 1600 mg kg^−1^ GMC groups, respectively, whereas 33, 75, and 58 OTUs were found in the three groups, respectively (Figure 5G). The principal coordinates analysis (PCoA), which is based on unweighted UniFrac, indicated that different dosages of GMC kept microbial composition away from the NCD group, which explained 14.68% of the total variance observed in PC1 (Figure 5H). In PC2, which explained 12.58% of the total variance, the gut microbiota in the NCD group separated from the 150 mg kg^−1^ GMC group but not from the 1600 mg kg^−1^ GMC group (Figure 5H). The analysis at the phylum level suggested that the gut microbiota in fecal samples was dominated by Firmicutes, Bacteroidetes, Actinobacteria, and Proteobacteria. Moreover, the 150 mg kg^−1^ GMC group showed that the relative abundance of Firmicutes increased while the relative abundance of Bacteroidetes decreased compared to the NCD group (Figure 5I). At the family level, there was a down-regulation in the abundance of S24–7 and an up-regulation in the abundance of Lactobacillaceae as a result of the 150 mg kg^−1^ GMC treatment. In contrast with the NCD group, the 1600 mg kg^−1^ GMC treatment group had lower levels of the abundance of Erysipelotrichaceae and higher levels of the abundance of family Clostridiales (Figure 5J).

### 3.6. Gut Microbiota Composition at Genus Level and Correlation Analysis of Blood Biochemical Criterion

**Figure 6:**
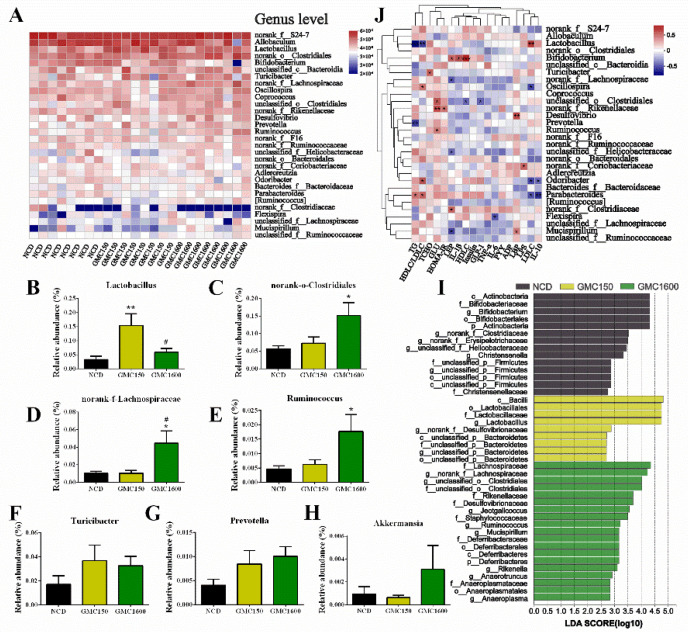
GMC supplementation changed the composition of intestinal microbiota. (**A**) A heat map of the relative abundance of the 30 most abundant genera among different treatments. The relative abundances of (**B**) Lactobacillus, (**C**) Clostridiales, (**D**) Lachnospiraceae, (**E**) *Ruminococcus*, (**F**) *Turicibacter*, (**G**) *Prevotella*, and (**H**) *Akkermansia* (not in top 30 genera). (**I**) LEfSe analysis among all the experimental groups (Log LDA > 2.0). (**J**) The correlation between the gut microbiota and blood biochemical criterion. Data are expressed as mean ± SEM, *n* = 8 (* *p* < 0.05 vs. NCD, ** *p* < 0.01 vs. NCD, # *p* < 0.05 vs. GMC150).

The relative abundance of the 30 most dominant genera in the three groups was analyzed. The results indicated that supplementing the diet with 150 mg kg^−1^ GMC significantly increased the level of Lactobacillus compared with the NCD group and the 1600 mg kg^−1^ GMC treatment group (*p* = 0.0096 and *p* = 0.0451, respectively, Figure 6A,B). Importantly, the addition of 1600 mg kg^−1^ GMC markedly increased the abundance of Clostridiales, Lachnospiraceae, and *Ruminococcus* compared to the NCD group (*p* = 0.0210, *p* = 0.0254, and *p* = 0.0443, respectively, Figure 6A,C–E) and had a higher relative abundance in *Turicibacter* and *Prevotella* than that in NCD group (Figure 6F,G). Changes in Akkermansia were also analyzed despite not being one of the 30 most abundant genera. Interestingly, the relative abundance of *Akkermansia* increased in the 1600 mg kg^−1^ GMC group (Figure 6H). Linear discriminant analysis effect size (LEfSe) comparison analysis was conducted to evaluate various dosages of GMC on the composition of gut microbiota (Figure 6I). The results indicated that class Bacilli, order Lactobacillales, family Lactobacillaceae, and *Lactobacillus* were dominant bacteria in 150 mg kg^−1^ GMC treatment group, and family Lachnospiraceae as well as order Clostridiales were advantage bacteria in the 1600 mg kg^−1^ GMC treatment group. In the NCD group, the domain bacteria were class Actinobacteria, order Bifidobacteriales, family Bifidobacteriaceae, and *Bifidobacterium*. The correlation between blood biochemical criterion and gut microbiota at the genus level was performed by Spearman’s correlation analysis (Figure 6J). Lactobacillus showed a strong positive correlation with LDL-C and strong negative correlations with TG as well as the ratio of HDL-C to LDL-C. Bifidobacterium was positively correlated with LEP, IL-1β, and HDL-C, while Lachnospiraceae was negatively correlated with LEP. Interestingly, Rikenellaceae had strong positive correlations with serum glucose and HOMA-IR.

### 3.7. GMC Supplementation Increased the Content of SCFAs in Feces

**Figure 7:**
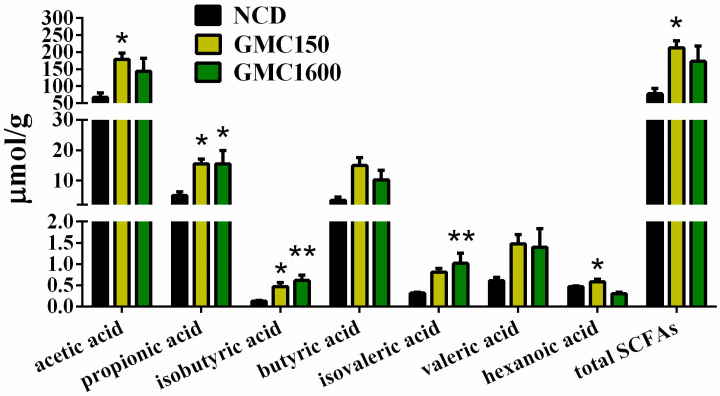
The concentrations of fecal SCFAs (acetic acid, propionic acid, isobutyric acid, butyric acid, isovaleric acid, valeric acid, hexanoic acid, and total SCFAs). Data are expressed as mean ± SEM, *n* = 8 (* *p* < 0.05 vs. NCD, ** *p* < 0.01 vs. NCD).

The concentration of SCFAs is shown in Figure 7. Supplementation of 150 mg kg^−1^ GMC resulted in obvious increases in most SCFAs compared to the NCD group, including acetic acid, propionic acid, isobutyric acid, hexanoic acid, and total SCFAs (*p* < 0.05). Similarly, the 1600 mg kg^−1^ GMC treatment group, in comparison with the NCD group, showed significantly increases in the contents of propionic acid, isobutyric acid, isovaleric acid, and hexanoic acid (*p* < 0.05).

## 4. Discussion

There is growing evidence that medium-chain fatty acid glyceride-based food additives can modulate the gut microbiota in mice and affect their health [14,15,16,17,18]. Originally, GMC was a medium-chain fatty acid glyceride food preservative. However, further studies are needed to clarify the specific effects of GMC on intestinal microbiota and host health. In the present research, we aimed to provide more practical and useful information on the application of GMC as a food preservative by studying its effect on C57BL/6 male mice with a normal diet. 

Notably, we found that GMC supplementation significantly decreased the relative weight of brown fat tissue and had a decrease in the relative weight of epididymal adipose and the size of epididymal adipocytes without affecting the body weight and feed intake. Interestingly, it is reported that medium-chain-triglyceride-fed rats had lighter fat pads [19]. In addition, we observed that the mice treated with 150 mg kg^−1^ GMC had a lower concentration of serum TG. These data collectively suggest that lipid metabolism in mice was affected by the intake of GMC. This could be explained by the fact that MCFAs are readily oxidized in the liver and lead to greater energy expenditure, thus resulting in decreased size of fat depots [20]. As members of the nuclear receptor superfamily, the roles of the peroxisome proliferator-activated receptors (PPARs: PPARα, PPARβ/δ, and PPARγ) in metabolic homeostasis were identified [21]. PPARα is known to modulate the expression of genes involved in the β-oxidation of fatty acids [22,23], such as its secondary target gene ACOX1, which is a rate-limiting enzyme in the β-oxidation of fatty acid [24,25]. Meanwhile, several studies have suggested that PPARα may increase fatty acid metabolism in the liver [26,27,28]. Here, our data showed that PPARα and ACOX1 expression was significantly up-regulated at a low dose in GMC-treated mice. Indeed, the decreased TG levels in mice were confirmed to be associated with the up-regulation of PPARα [29]. These data collectively imply that 150 mg kg^−1^ GMC might increase the metabolism of fatty acids. PPARγ, is also expressed in the liver, despite being predominantly expressed in adipose [30]. Previous studies have demonstrated that the increased expression of PPARγ in adipose tissue is responsible for fat accumulation [31]. In contrast, other studies have shown that decreased lipids in the liver are associated with up-regulation of PPARγ [30,32]. Therefore, more research may be needed on such a controversial point. CD36 is transcriptionally regulated by PPARγ and also plays a role in regulating fatty acids metabolism [33]. Here, an evident up-regulation of PPARγ2 and CD36 was observed after 1600 mg kg^−1^ GMC treatment. This may be consistent with the reduction of the lipid by the GMC treatment. HMGCR is a rate-limiting enzyme in the synthesis of cholesterol by hepatocytes [34]. Here, we showed that HMGCR was up-regulated and consistent with the decline in the ratio of HDL-C to LDL-C after GMC high-dose treatment, which reminds us that the effect of GMC on lipid metabolism needs further study. Moreover, PRDM16, which is a transcriptional regulator in the differentiation of brown adipocyte, had a significantly higher expression in the 1600 mg kg^−1^ GMC group [35]. Meanwhile, UCP1, the regulatory gene of BAT-thermogenesis, also had a slightly increased level in the high-dose GMC treatment group [36]. These data indicate that 1600 mg kg^−1^ GMC may have a positive effect on stimulating the differentiation and thermogenesis of brown fat.

Disturbed homeostasis of glucose metabolism is one of the dominant features of metabolic syndrome and has a high risk for the development of some metabolic diseases, such as obesity and type 2 diabetes [37,38]. Thus, it is important to evaluate the effect of GMC on glucose metabolism. G6PC and PEPCK are two key gluconeogenic enzymes in hepatocytes, highly activated during fasting and suppressed in the fed state by insulin [39]. The up-regulation of G6PC and PEPCK in the low-dose GMC treatment group was in line with the significantly increased protein expression of FOXO1, which plays a vital role in mediating the effects of insulin on glucose metabolism [40]. Moreover, serum insulin decreased slightly with 150 mg kg^−1^ GMC treatment, consistent with the obvious down-regulation of GLP-1, which is known to potentiate insulin secretion [41]. These data suggest that 150 mg kg^−1^ GMC may play a regulatory role in glucose homeostasis through gluconeogenesis. CHREBP is one of the major transcription factors that regulate carbohydrate metabolism; LPK, which is the target gene of CHREBP, also plays an important role in glycolysis [42]. Similarly, GCK, which is the key factor in glycolysis, can also regulate insulin secretion and glucose metabolism of the liver, and loss of its activity can lead to diabetes [43]. We speculated that 1600 mg kg^−1^ GMC may have an effect on glycolysis to regulate glucose metabolism. Referring to the above results, the supplementation of GMC had no adverse effects on glucose metabolism in mice, and these findings are similar with our previous study [16]. 

TLR2, which belongs to pattern-recognition receptors, is regarded as the major cause of sustaining inflammation [44]. The expression of TNF, as well as MCP-1, also plays a crucial role in inflammatory response. Here, the mRNA levels of TLR2, TNF, and MCP-1 were decreased by GMC treatment. In addition, evidence suggests that the infiltration of mouse macrophages in the liver is mainly controlled by MCP-1, whereas IL-10 regulated the inflammatory response by inhibiting the release of inflammatory mediators by mononuclear macrophages [45,46]. In our study, we observed that the serum content of pro-inflammatory cytokines MCP-1 and IL-1β was decreased in the 1600 mg kg^−1^ GMC group in comparison with the NCD group, whereas the anti-inflammatory cytokine IL-10 had a significant increase in the 150 mg kg^−1^ GMC group. Given the effect of GMC on the inflammatory response, we hypothesized that GMC might have the potential to improve inflammation.

Accumulated research has revealed that intestinal microbiota composition and structure play crucial roles in host health and are associated with a variety of diseases [47]. Intestinal microbes interact with their hosts and depend on each other. In other words, the host can provide nutrients for gut microbes, which in turn help the host digest dietary fiber to produce SCFAs [48]. In the present study, we found that the α-diversity of gut microbiota significantly increased after GMC treatment. Moreover, the Venn diagram demonstrated that OTUs in the GMC supplementation groups increased. Recent research performed by Nishida et al. revealed that the decreased diversity of gut microbiota plays a vital role in the occurrence of inflammatory bowel disease [49]. Hence, increased diversity may be a desired health benefit as it maintains gut microbiota balance. Additionally, the β-diversity indicated that the samples in the GMC group were clustering intensively and gradually away from the NCD group, which suggested communities of gut microbiota altered by GMC treatment in a particular direction. Recently, growing evidence has suggested that the abundance of Firmicutes in the intestine decreases, and the abundance of Bacteroidetes increases, in some patients with severe diseases such as sepsis, cirrhosis, and Alzheimer’s disease [50,51,52]. In support of this, Xu et al. considered that disease severity had negative correlations with Firmicutes, Clostridia, and Ruminococcaceae abundances [53]. Zhao et al. also found that there was an increase in the abundance of Firmicutes and a decrease in the abundance of Bacteroidetes after GML treatment [15]. Moreover, it has been identified that *Akkermansia* has positive effects on host health [54]. In our research, the abundance of Firmicutes was increased, while the abundance of Bacteroidetes was decreased, in the 150 mg kg^−1^ GMC group. Additionally, the abundance of Clostridiales and *Ruminococcus* significantly increased under 1600 mg kg^−1^ GMC treatment and the abundance of *Akkermansia* also saw an increase. These results together suggest that the addition of GMC may contribute to the maintenance of host health. *Lactobacillus*, as a recognized probiotic, is increasingly used in food and medicine to balance the disturbed intestinal microbiota and related gastrointestinal dysfunction [55,56,57]. Notably, the significantly increased abundance of *Lactobacillus* in the low-dose group also suggests the benefits of GMC to host health, which is consistent with our previous study on GML [15]. Moreover, with a GMC treatment of 1600 mg kg^−1^, SCFA producers (Clostridiales, Lachnospiraceae, and *Ruminococcus*) were significantly increased, while *Prevotella* and *Turicibacter* were slightly increased. These bacteria are major producers of butyrate, which can increase the amount and enhance the function of regulatory T cells [58,59,60,61]. The rise of SCFA producers was consistent with our observations of the significantly increased SCFAs extracted from feces in GMC groups. It is universally acknowledged that SCFAs are the mediator of the interaction between intestinal microbiota and host metabolism and are associated with host health [48,62]. The increase of acetic acid may be one of the reasons for the TG decline in the 150 mg kg^−1^ treatment [11]. A review that summarized current studies on the effects of SCFAs on human health indicated that SCFAs can enter the systemic circulation to affect surrounding tissues, improve blood glucose and insulin sensitivity, and have the function of preventing obesity and related diseases [63]. Notably, it was observed that the two doses of GMC had the same effect of on the content of SCFAs. We speculated that the low dose of GMC would have a significant effect on gut microbes, in particular stimulating the production of SCFAs by certain gut microbiota. Thus, with the increase of GMC concentration, the content of SCFAs did not result in more significant changes. Collectively, in our research, the increased abundance of SCFA producers and the consequent increase in the concentration of SCFAs showed that the addition of GMC may play a crucial role in maintaining the health of the host and may alleviate the occurrence of certain diseases.

## 5. Conclusions

The present study indicated that GMC supplementation significantly modulated the gut microbiota composition and significantly increased the abundance of SCFA producers, resulting in the rise of SCFA content. Furthermore, most of the metabolism-related indicators and inflammatory cytokines were not adversely regulated by GMC. The current study comprehensively assessed the impacts of GMC on host health from multiple perspectives, such as intestinal microecology, glucose and lipid metabolism, and inflammation, providing a new scientific basis for the application of GMC in the food industry.

## Data Availability

Data described in the manuscript will be provided on reasonable request to the corresponding author.

## Supplementary Materials

The following are available online at https://www.mdpi.com/article/10.3390/nu13051427/s1, Figure S1: H&E staining of liver in each group, Table S1: Primer sequences used for qRT-PCR analysis.

## Abbreviations

GMC	glycerol monocaprylate
MCFA	medium-chain fatty acid
SCFA	short-chain fatty acid
GML	glycerol monolaurate
TG	triglyceride
HDL-C	high-density lipoprotein cholesterol
LDL-C	low-density lipoprotein cholesterol
FFA	free fatty acid
LEP	leptin
PYY	peptide YY
GLP-1	glucagon like peptide 1
ADP	adiponectin
LBP	lipopolysaccharide binding protein
LPS	lipopolysaccharide
IL-1β	interleukin 1beta
IL-6	interleukin 6
IL-10	interleukin 10
TNF	tumor necrosis factor
MCP-1	monocyte chemotactic proteins 1
H&E	haematoxylin and eosin
PPARα	peroxisome proliferators activate receptor alpha
PPARγ2	peroxisome proliferators activate receptor gamma2
SCD1	stearoyl-CoA desaturase 1
CYP7A1	cholesterol 7 alpha-hydroxylase A1
SREBP-1C	sterol regulatory element-binding protein-1c
HMGCR	3-hydroxy-3-methylglutaryl-coenzyme A reductase
G6PC	glucose-6-phosphatase catalytic subunit
PEPCK	phosphoenolpyruvate carboxykinase
GCK	glucokinase
TLR2	toll-like receptor 2
MCP-1	monocyte chemotactic protein-1
CD36	cluster of differentiation 36
FASN	fatty acid synthase
ACOX1	acyl-coenzyme A oxidase 1
FGF21	fibroblast growth factor 21
CHREBP	carbohydrate responsive element binding protein
LPK	L-typepyruvatekinase
PRDM16	PR domain-containing 16
UCP1	uncoupling protein 1
FOXO1	forkhead box transcription factor O1
YWHAZ	tyrosine 3-monooxygenase/tryptophan 5-monooxygenase activation protein zeta
