# Supplementary material for: Glycerol Monocaprylate Modulates Gut Microbiota and Increases Short-Chain Fatty Acids Production without Adverse Effects on Metabolism and Inflammation
Source: Nutrients. 2021 Apr 23;13(5):1427. doi: 10.3390/nu13051427 (PMC8147114; doi:10.3390/nu13051427)
Supplement: Supplementary file 1 [file nutrients-13-01427-s001.zip › nutrients-1152147-supplementary.pdf]

nutrients

Glycerol Monocaprylate Modulates Gut Microbiota and Increases Short-Chain Fatty Acids Production Without Adverse Effects on Metabolism and Inflammation

Junhui Zhang [1,2], Fengqin Feng [1,2] and Minjie Zhao [1,2,*]

1 College of Biosystems Engineering and Food Science, National Engineering Laboratory of Intelligent Food Technology and Equipment, Key Laboratory for Agro-Products Postharvest Handling of Ministry of Agriculture and Rural Affairs, Key Laboratory for Agro-Products Nutritional Evaluation of Ministry of Agriculture, Zhejiang University, Hangzhou 310058, China; zhangjunhui9916@163.com (J.Z.); feng_fengqin@hotmail.com (F.F.)
2 Ningbo Research Institute, Zhejiang University, Ningbo 315100, China
* Correspondence: minjiezhao@zju.edu.cn; +86-571-88982981

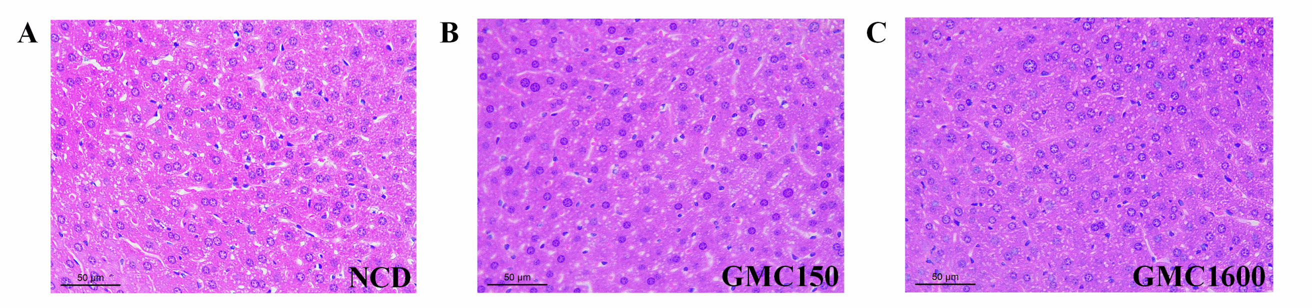

Figure S1. H&E staining of liver in each group.

Table S1. Primer Sequences Used for qRT-PCR Analysis.

	Forward Primer (5'-3')	Reverse Primer (5'-3')
PPARα	GTCCTCAGTGCTTCCAGAGG	GGTCACCTACGAGTGGCATT
PPARγ	GCATTTCTGCTCCACACTATGA	TCGCACTTTGGTATTCTTGG
SCD1	CCGAAGAGGCAGGTGTAGAG	TTCTTACACGACCACCACCA
CYP7A1	TGGGCATCTCAAGCAAACAC	TCATTGCTTCAGGGCTCCTG
SREBP-1C	GATCAAAGAGGAGCCAGTGC	TAGATGGTGGCTGCTGAGTG
HMGCR	GGACCAACCTTCTACCTC	CCATCACAGTGCCACATAC
G6PC	TCTGTCCCGGATCTACCTTG	GTAGAATCCAAGCGCGAAAC
PEPCK	TAGGAGCAGCCATGAGAT	CGAAGTTGTAGCCGAAGA
GCK	TCCCTGTAAGGCACGAAGAC	ACGATGTTGTTCCCTTCTGC
TLR2	CACCGGTCAGAAAACAACTTACC	CAAGATCCAGAAGAGCCAAAGAG
TNFα	AGGCACTCCCCCAAAAGAT	CAGTAGACAGAAGAGCGTGGTG
MCP-1	GTGCTGACCCCAAGAAGGAA	GTGCTGAAGACCTTAGGGCA
YWHAZ	TTCTTGATCCCCAATGCTTC	TTCTTGTCATCACCAGCAGC
UCP1	GGTGTGGCAGTGTTCATT	TGGTGGCTATAACTCTGTAAG
PRDM16	TGGCTGAGACCTACTGAG	AGACAATGGCTGGAATGG
FGF21	CTGGAGGACGGTTACAATG	GAAGAGTCAGGACGCATAG
CHREBP	CCTTAGTTCCTGAGCAGAG	CAGTCACACCAACACCAA
LPK	CTCCCCACTCAGCTACAGACC	CCCTTCACAATTTCCACCTCC
CD36	GCTTGCAACTGTCAGCACAT	GCCTTGCTGTAGCCAAGAAC
ACOX1	CTATGGGATCAGCCAGAAAGG	AGTCAAAGGCATCCACCAAAG
FASN	AGGGGTCGACCTGGTCCTCA	GCCATGCCCAGAGGGTGGTT
